# Identification of potential ferroptosis-related biomarkers in proliferative vitreoretinopathy based on machine learning

**DOI:** 10.3389/fmolb.2026.1725407

**Published:** 2026-02-10

**Authors:** Jiangying Liu, Shengxiang Zhang, Lingdan Wu, Yingchao Xue, Boyu Liu, Hongyan Song, Qihua Xu

**Affiliations:** 1 School of Optometry, Jiangxi Medical College, Nanchang University, Nanchang, Jiangxi, China; 2 Department of Ophthalmology, The Affiliated Eye Hospital, Jiangxi Medical College, Nanchang University, Nanchang, Jiangxi, China; 3 Jiangxi Research Institute of Ophthalmology and Visual Science, Nanchang, Jiangxi, China; 4 Jiangxi Provincial Key Laboratory for Ophthalmology, Nanchang, Jiangxi, China

**Keywords:** bioinformatics, biomarkers, ferroptosis, immune infiltration, machine learning, proliferative vitreoretinopathy

## Abstract

**Background:**

Proliferative vitreoretinopathy (PVR) is a blinding retinal condition often linked to retinal detachment, eye trauma, and complications following intraocular surgery. Although oxidative stress and epithelial–mesenchymal transition (EMT) are well-recognized contributors to PVR pathogenesis, whether ferroptosis-related pathways are involved in this process remains unclear.

**Methods:**

Differentially expressed genes (DEGs) were identified from the GSE28133 dataset and intersected with ferroptosis-related genes curated from FerrDb to obtain ferroptosis-related differentially expressed genes (FRDGs). Functional enrichment analyses, protein–protein interaction network construction, and machine learning approaches (LASSO regression and SVM-RFE) were applied to identify key candidate genes. Immune infiltration was analyzed using CIBERSORT. Experimental validation was performed using an *in vitro* EMT model of retinal pigment epithelial cells and a rabbit PVR model.

**Results:**

Functional enrichment analyses indicated that FRDGs were mainly involved in wound healing, tissue remodeling, oxidative stress responses, and ferroptosis-related pathways. TIMP1 and STAT3 were identified as ferroptosis-associated candidate genes with strong discriminative ability between PVR and control samples in the discovery dataset. Immune infiltration analysis revealed distinct immune cell profiles in PVR samples and significant correlations between TIMP1 and STAT3 expression and multiple immune cell subsets. Experimental validation confirmed upregulation of TIMP1 and STAT3 in the EMT model. In addition, Western blot analysis demonstrated significantly increased GPX4 protein expression in retinal tissues from the rabbit PVR model compared with controls.

**Conclusion:**

This study identifies TIMP1 and STAT3 as ferroptosis-associated candidate genes in proliferative vitreoretinopathy and highlights potential links among ferroptosis-related regulatory pathways, immune microenvironment alterations, and PVR pathogenesis. These findings provide a foundation for further mechanistic studies to clarify the role of ferroptosis in PVR.

## 1 Introduction

Proliferative vitreoretinopathy (PVR) is a blinding disease of the fundus of the eye that commonly occurs after retinal detachment (RD), ocular trauma, and internal eye surgery. PVR is considered to be a long-lasting and amplified scar repair process, and its basic pathological process includes cell migration, cell proliferation, extracellular matrix production, and ultimately the formation of a contractible, avascular, fibroblastic proliferative membrane in the vitreous cavity and peripapillary retinal area (Hilton et al., 1983; Mudhar, 2020), and at present, surgery is still the main treatment for PVR (Coffee et al., 2014).

Current studies have shown that retinal pigment epithelium (RPE) cells are one of the key cells in the development of PVR. Under normal physiological conditions, the differentiated and mature RPE cells remain in a quiescent and non-dividing state, which plays an important role in maintaining the normal function of the retina and choroid. However, when retinal detachment occurs due to retinal lacunae or trauma to the eye, a variety of factors lead to the disruption of the connective complex of RPE cells, which separate from Bruch’s membrane and migrate to the retinal neuroepithelial defects, where they undergo hyperplasia and transform into myofibroblasts to form a fibro-proliferative membrane, which ultimately exacerbates retinal detachment and leads to a further decline in visual acuity. During this process, RPE cells undergo epithelial–mesenchymal transition (EMT), transforming from epithelial to mesenchymal cells, which enhances their proliferation, migration, and resistance to apoptosis, and secrete more extracellular matrix, which plays a key role in the formation of PVR (Tamiya and Kaplan, 2016; Chen et al., 2015). The molecular mechanisms underlying the development of PVR have not yet been fully clarified, and therapeutic options are still limited. PVR, as a class of clinical complications that require long-term control, seriously affects the physical and mental health and quality of life of patients and imposes a huge economic burden on them, so progress in elucidating the pathogenesis of PVR may be the basis for the development of therapeutic approaches to PVR.

Ferroptosis is a new modality of programmed cell death that differs from apoptosis and involves high-iron-dependent lipid peroxidation (Qiu et al., 2020). The accumulation of lipid peroxides leads to a loss of selective cell membrane permeability (Stockwell et al., 2017). However, glutathione peroxidase 4 (GPX4) plays an important role in protecting cell membranes from peroxidative damage (Yang et al., 2014), which protects cell membranes from the effects of oxidation. When ferroptosis occurs, the antioxidant glutathione is depleted, leading to the failure of GPX4, which ultimately leads to the fatal accumulation of lipid peroxides (Stockwell et al., 2020). Recent studies have suggested that ferroptosis-related pathways can interact with oxidative stress and EMT in various disease contexts (Ren et al., 2023; Yu et al., 2021). However, whether ferroptosis-related regulatory mechanisms are involved in PVR remains largely unexplored, and direct evidence for ferroptotic cell death in PVR is currently lacking.

In this study, we performed an integrative transcriptomic analysis to explore ferroptosis-associated gene expression patterns in PVR. By combining public microarray data, bioinformatic analyses, machine learning approaches, immune infiltration analysis, and experimental validation, we aimed to identify ferroptosis-associated candidate genes and to investigate their potential relevance to PVR-associated pathological processes.

## 2 Materials and methods

### 2.1 Data acquisition and processing

**FIGURE 1:**
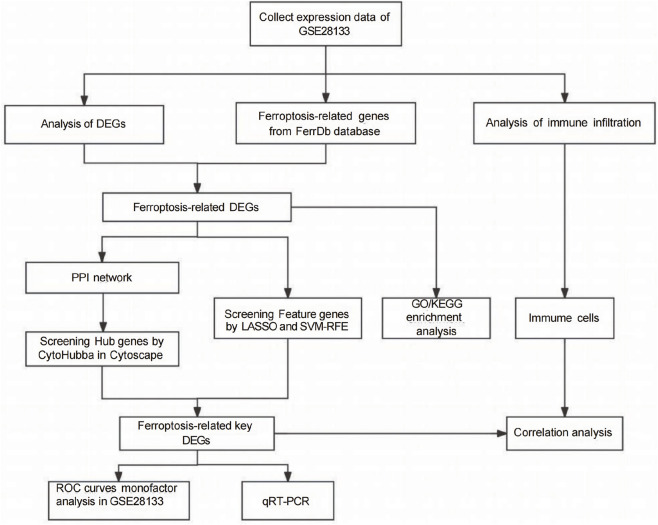
The flowchart of the analysis process.

As shown in Figure 1, The eligible expression dataset (GSE28133) was searched and downloaded using the Gene Expression Omnibus (GEO) database. This dataset includes retinal tissue samples from 19 retinal detachment patients with PVR and 19 retinal samples from healthy individuals. The data were analyzed using R software (version 4.3.2) (Yao et al., 2020). The gene probe IDs were converted to gene symbols using R software according to the GPL570 platform of Affymetrix Human Genome U133 Plus 2.0 Array. The probes were matched to genes from the GPL570 platform for microarray annotation. The public FerrDb (http://www.zhounan.org/ferrdb) database was searched for genes related to promoting, inhibiting, labeling or unclassified ferroptosis, and after removing duplicate genes, 564 ferroptosis-related genes were finally obtained for subsequent analysis.

### 2.2 Identification of DEGs and ferroptosis-related DEG

DEGs were identified using the Limma package of R software. Genes with an adjusted p-value (false discovery rate, FDR) < 0.05 and |log2 fold change| > 1 were defined as DEGs, and finally, a volcano plot was constructed using the “ggplot 2” package to visualize the results.

We intersected 564 ferroptosis-related genes with DEGs to obtain ferroptosis-related differential genes (FRDGs). A Venn diagram was constructed using the Bioinformatics Visualization Cloud Platform (URL: https://www.bioladder.cn) to visualize the results.

### 2.3 Functional enrichment analysis of FRDGs

To investigate the potential biological roles of FRDGs in more detail, the “ClusterProfiler” package (Yu et al., 2012) (version: 3.18.0) and “org.Hs.e.g.,.db” package in R software were utilized to perform Gene Ontology (GO) function enrichment analysis and Kyoto Encyclopedia of Genes and Genomes (KEGG) pathway enrichment analysis, with q value <0.05 as the enrichment threshold. GO analysis was primarily used to annotate gene functions, especially biological pathways (BP), cellular components (CC) and molecular functions (MF). KEGG analysis was used to detect pathway enrichment in FRDGs (Kanehisa et al., 2025; Kanehisa, 2019; Kanehisa and Goto, 2000).

### 2.4 Protein-protein interaction (PPI) network construction and screening of hub genes

The STRING database was utilized to observe interactions between the ferroptosis-related DEGs. The confidence score is 0.4. Cytoscape software (version 3.10.1) was used to construct and visualize the PPI network. Using the cytohubba plugin, the hub genes in the PPI network were computed by five algorithms, namely, Maximum Cluster Centrality (MCC), Maximum Neighborhood Component (MNC), Degree, Edge Discrete Component (EPC), and Closeness.

### 2.5 Screening genes using machine learning algorithms

Two machine-learning algorithms were used to predict disease status to identify significant feature genes. LASSO is a regression analysis algorithm that improves prediction accuracy through regularization. As a popular supervised machine-learning technique, the Support vector machine (SVM) is frequently applied for classification and regression. To avoid overfitting, the optimal genes were selected from the metadata cohort using an RFE algorithm (Bao et al., 2023). The LASSO regression and SVM-RFE were applied to screen the feature genes. The genes obtained by the two methods were taken to be intersected; the overlapping ones were the feature genes.

### 2.6 Screening ferroptosis-related key DEGs (FRKGs)

Hub genes obtained by PPI network construction and feature genes obtained by screening using machine learning algorithms, both of them take the overlapping genes, and the overlapping genes we consider to be the ferroptosis-related key DEGs in PVR.

### 2.7 Rabbit PVR model and experimental validation

We established rabbit animal models of proliferative vitreoretinopathy and then performed high-throughput sequencing to observe the changes in gene expression levels. Adult rabbits were purchased from Ganzhou Animal Husbandry and Aquatic Research Institute. Rabbits were anesthetized with intramuscular ketamine hydrochloride (40 mg/kg, Gutian Pharmaceutical Co., Fujian, China) and then with intravenous pentobarbital sodium (50 mg/kg, Sinopharm Chemical Reagent Co.,Shanghai, China). Rabbits were euthanized in the experiment by intravenous overdose of pentobarbital sodium (100 mg/kg, Sinopharm Chemical Reagent Co. Shanghai, China). Eyelid opening device was used to open the eyes, 5 mm of the corneosclera margin above the temporal layer, resulting in scleral puncture orificium about 3 mm long. 0.3 mL vitreous body was carefully sucked out with a 1 mL syringe, and 0.3 mL platelet-rich plasma (PRP) was injected into the vitreous cavity with a vitreous syringe needle (Wu et al., 2022). This trial was reviewed and approved by the Medical Ethics Committee of the Affiliated Eye Hospital of Nanchang University (approval Number: YLP20221206). All experiments were conducted in accordance with the relevant designated guidelines and regulations and in compliance with the ARRIVE guidelines.

RNA-seq libraries were prepared using NEBNext Ultra II Kit (Illumina). Sequencing was performed on NovaSeq 6,000. Reads were aligned to OryCun2.0 using HISAT2 (v2.1.0), and gene expression quantified via htseq-count (v0.11.2). Differential analysis used DESeq2 (v1.22.2) with thresholds |log2FC| > 1 and adj. p < 0.05.

For Western blotting, retinal tissues were homogenized in ice-cold RIPA lysis buffer supplemented with protease and phosphatase inhibitor cocktails. Lysates were clarified by centrifugation (12,000 × g, 15 min, 4 °C), and protein concentration was determined using a BCA assay. Equal amounts of protein (20–40 μg per lane) were separated by SDS–PAGE and transferred onto PVDF membranes. Membranes were blocked with 5% non-fat milk in TBST for 2 h at room temperature and incubated overnight at 4 °C with primary antibodies against GPX4(Proteintech 67763-1-Ig, 1:1000) and GAPDH(Proteintech 60004-1-Ig, 1:20000). After washing, membranes were incubated with HRP-conjugated secondary antibodies for 1 h at room temperature. Protein bands were visualized using enhanced chemiluminescence (ECL) and imaged with a chemiluminescence detection system.

### 2.8 Dataset validation of FRKGs

The GSE28133 dataset was validated for FRKGs by performing a one-way analysis of subject operating characteristic (ROC) curves. GraphPad Prism software was used to plot the ROC curves. Any gene with an area under the ROC curve of >0.9 was considered to be of high diagnostic value.

### 2.9 Immune infiltration analyses

The CIBERSORT package (Newman et al., 2015) was utilized to analyze immune cell infiltration in PVR and normal samples using the GSE28133 dataset. Then normalized gene expression data was transformed into immune cell information by the CIBERSORT deconvolution algorithm. Linear regression analysis was used to analyze the correlation between FRKGs expression and immune cells. A p-value 0.05 was considered statistically significant. The results were visualized using the ggplot2 pack.

### 2.10 Cell culture and modeling of EMT

All cell experiments were conducted using ARPE-19 cells, an immortalized retinal pigment epithelium cell line derived from normal adult humans, were purchased from Procell Co. (CL-0026). ARPE-19 cells were cultured in DMEM/F12 medium supplemented with 10% fetal bovine serum (FBS) and 1% penicillin-streptomycin, and placed in an incubator at 37 °C under saturated humidity with 5% CO_2_ for incubation. The EMT model was established by treating RPE cells with TGF-β2 (Concentration of 10 ng/mL).

### 2.11 Quantitative real-time polymerase chain reaction (qRT-PCR) analysis

For each sample, 500 μL of vitreous fluid was collected for RNA extraction. Total RNA was extracted using TRIzol Reagent (Shanghai Sangyo, Shanghai, China). Subsequently, RNA concentration was detected using Qubit2.0, and the integrity of RNA and its genomic contamination were assessed by agarose gel electrophoresis. Total RNA was extracted, reverse transcribed into cDNA templates, and PCR amplified according to the instructions. The total RNA was extracted, reverse transcribed into cDNA templates, and amplified by PCR according to the instructions. Based on the Ct values of each group, GA was selected as the internal reference gene, and the relative expression of mRNA in each group was calculated by the 2^−ΔΔCT^ method. The primers used in this study are detailed in Table 1. Independent sample t-tests were then performed using GraphPad Prism nine software, and p < 0.05 was considered to indicate a significant difference.

**TABLE 1:** Primers Used for qRT-PCR in This Study.

Gene	Forward primer (5′-3′)	Reverse primer (5′-3′)
STAT3 TIMP1	CATCCTGAAGCTGACC CAGGTTGGCTGTGAGG AATGCACA	TCCTCACATGGGGGAG GTAGGTCCACAAGCAA TGAGTGCC

### 2.12 Statistical analysis

All data were processed and studied using Excel and GraphPad Prism 8 software. Comparisons between the two groups were made using the independent samples t-test. p < 0.05 values were statistically significant.

All R scripts used for data preprocessing, differential expression analysis, enrichment analysis, protein–protein interaction network construction, machine learning, and immune infiltration analysis are publicly available at: https://github.com/liujiangying68/PVR-ferroptosis-transcriptomics.

## 3 Results

### 3.1 Identification of DEGs

**FIGURE 2:**
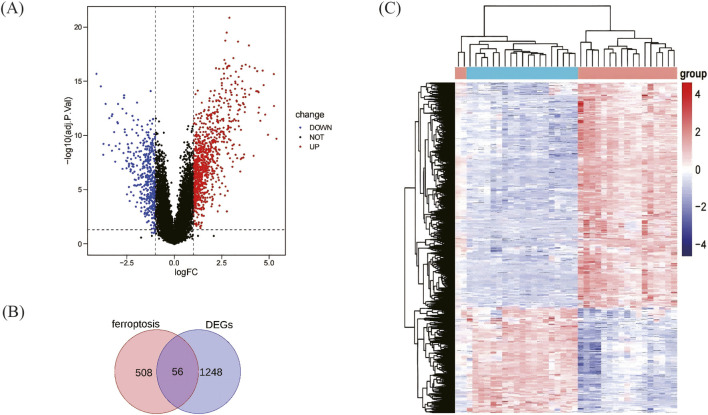
Analysis of differentially expressed genes (DEGs). **(A)** DEGs diagram of volcano. **(B)** The Venn diagram of ferroptosis -related DEGs. **(C)** DEGs heat map: Blue represents the normal group, and red represents the PVR group.

Differential gene analysis was performed using genes from 19 retinal tissues of retinal detachment patients with PVR and 19 normal retinal tissues. An adjusted p-value <0.05 and | logFC| > 1 were used as criteria. A total of 1304 DEGs were identified, including 890 upregulated genes and 414 downregulated genes. The differential expression of these 1304 DEGs in the PVR group and the control group is shown in the volcano diagram (Figure 2A), A heat map of these DEGs revealed variations in relative gene expression among the PVR and normal samples (Figure 2C).

### 3.2 Identification of ferroptosis-related DEGs (FRDGs)

The FRDGs were identified by taking the intersection of the ferroptosis-related gene data from the ferrDb database with the differential genes. 46 upregulated genes and 10 downregulated genes were identified after removing duplicates (Figure 2B; Table 2). The roles of ferroptosis-related differentially expressed genes in ferroptosis are shown in (Table 3). 32 out of 348 genes in the ferroptosis suppressors were detected, 18 out of 369 genes in the ferroptosis drivers were detected and 2 out of 11 genes in the ferroptosis markers were detected.

**TABLE 2:** A list of 56 differentially expressed ferroptosis-related genes.

Expression	Number	Gene
Up	46	TIMP1, FZD7, FTL, PARP9, CTSB, TMSB4X, HSD17B11, CAPG, CAV1, CP, RGS4, TP53, STAT3, DECR1, DDR2, PARP14, TRIM21, CD44, CDKN1A, HAMP, RBMS1, ANO6, QSOX1, RELA, CBR1, RARRES2, PARP12, HMOX1, SMPD1, FXN, NUPR1, PANX1, ENPP2, IDH1, NNMT, PIR, PTPN6, WWTR1, PLIN2, AKR1C3, HSPB1, NCF2, YAP1, P4HB
Down	10	GJA1, TXNIP,DRD4, GOT1, NOX4, SLC40A1, NR4A1, AGPAT3, DUSP1, MEF2C

**TABLE 3:** Roles of ferroptosis-related differentially expressed genes in ferroptosis.

Category	Gene
Ferroptosis drivers	GOT1, AGPAT3, TP53, ANO6, CTSB, QSOX1, TIMP1, PANX1, SMPD1, NOX4, IDH1, PTPN6, WWTR1, TRIM21, YAP1, HMOX1, DDR2, GJA1
Ferroptosis suppressors	HSPB1, FZD7, CD44, CDKN1A, ENPP2, CAV1, SMPD1, GOT1, PIR, P4HB, PARP9, RARRES2, SLC40A1, TP53, AKR1C3, TMSB4X, DECR1, PLIN2, RELA, FTL, FXN, PARP14, NUPR1, HMOX1, STAT3, MEF2C, PARP12, PPARA, SLC16A1, RBMS1, NR4A1 CP
Ferroptosis markers	HSPB1, SLC40A1

### 3.3 Functional enrichment analyses of FRDGs

**FIGURE 3:**
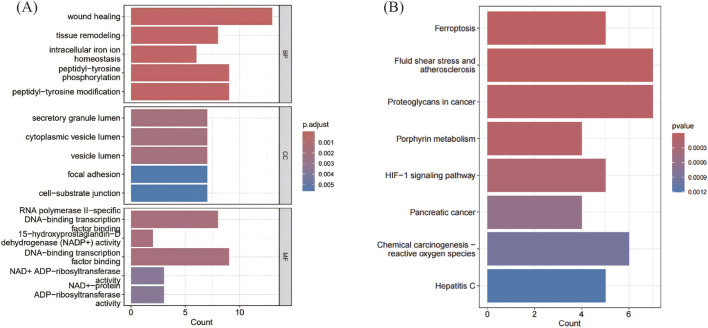
Functional enrichment analysis of FRDGs. **(A)** The top significantly enriched GO terms in the category of biological process, cellular component, and molecular function for the FRDGs. **(B)** KEGG pathway enrichment analysis for the FRDGs.

We performed GO and KEGG enrichment analyses using R software to detect potential biological activities of FRDGs. According to the results of GO analysis, the most important enrichment terms were wound healing, tissue remodeling, and cellular iron homeostasis (Figure 3A). KEGG pathway analysis showed that these FRDGs were significantly enriched in ferroptosis, reactive oxygen species, atherosclerosis, and HIF-1 signaling pathways (Figure 3B).

### 3.4 PPI network construction and screening of hub genes

**FIGURE 4:**
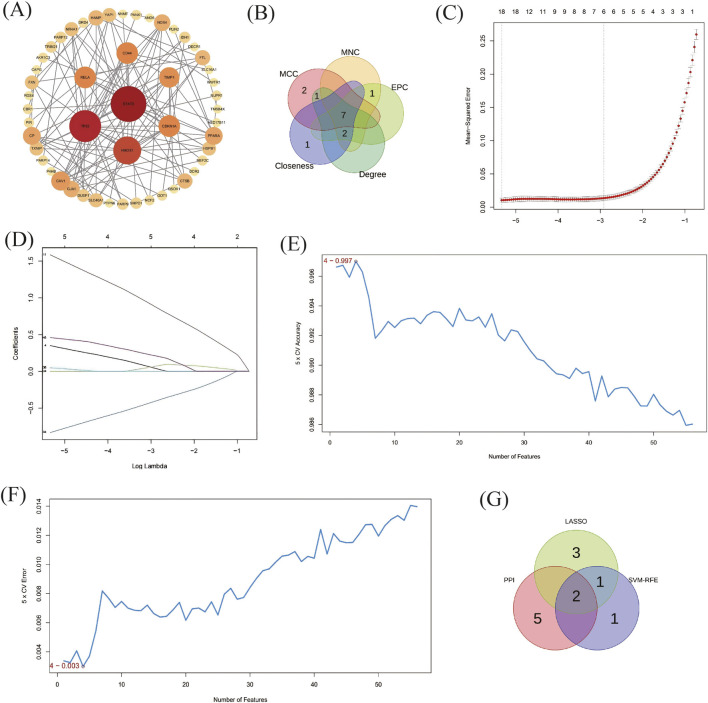
Protein- Protein interaction (PPI) analysis, Identification of genes based on machine learning and screening of ferroptosis-related key DEGs (FRKGs). **(A)** The PPI network of FRDGs. **(B)** Screening of hub genes by Five algorithms (MCC, MNC, Degree, EPC, Closeness) **(C,D)** Least absolute shrinkage and selection operator (LASSO) regression algorithm to screen six FRDGs. **(E,F)** SVM-RFE was used to select four FRDGs. **(G)** The FRKGs obtained from the PPI, LASSO and SVM-RFE models.

A protein-protein interaction network was constructed to demonstrate the interactions between FRDGs (Figure 4A). We obtained a PPI network with 120 nodes and 51 edges. 4 out of 56 genes are not related to other molecules and do not form a molecular network. 43 upregulated genes and nine downregulated genes are in the PPI network. Five algorithms (MCC, MNC, Degree, EPC, Closeness) were used to obtain the top 10 hub genes, and the overlapping genes in these five algorithms were chosen as the hub genes. The hub genes we obtained were CDKN1A, HMOX1, CD44, CAV1, STAT3, TIMP1, TP53; all of them were upregulated genes (Figure 4B).

### 3.5 Screening genes using machine learning algorithms and acquisition of FRKGs

LASSO regression was applied to all 56 FRDGs. six genes were screened from LASSO regression algorithm (Figure 4CD), which were CP, TIMP1, FZD7, DRD4, STAT3, FTL. four genes could be screened from SVM-RFE algorithm (Figure 4EF), which were TIMP1, FZD7, PARP9, STAT3. genes obtained from the two machine learning methods were intersected with the pivotal genes obtained from PPI. A total of two ferroptosis-related key DEGs (FRKGs) were obtained, which were TIMP1, STAT3 (Figure 4G).

### 3.6 Dataset validation of FRKGs

**FIGURE 5:**
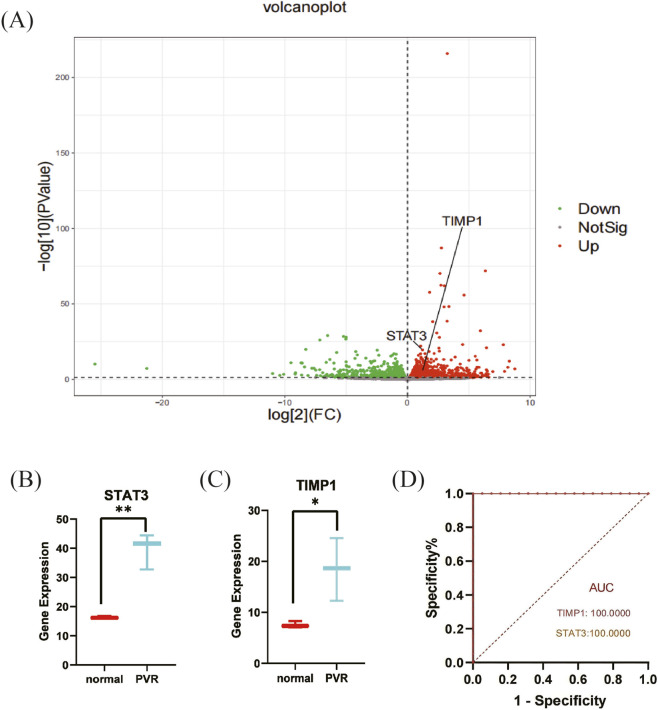
Database validation of ferroptosis-related key DEGs (FRKGs). **(A)** Volcano **(B)** Gene expression of STAT3. **(C)** Gene expression of TIMP1. **(D)** ROC curve of FRHGs in the GSE28133 dataset. PVR, Proliferative vitreoretinopathy.

Through the high-throughput sequencing results of rabbit animal models (Supplementary Material), we found that expression of FRKGs was higher in the PVR samples than in the normal samples (p < 0.05) (Figures 5A–C), which is consistent with the results of the GSE28133 dataset; the ferroptosis-related key DEGs were analyzed by one-way analysis of ROC curves. The results showed that the diagnostic accuracy of TIMP1 and STAT3 for PVR were both 100.00% in the GSE28133 dataset (Figure 5D); this suggests that TIMP1 and STAT3 have great diagnostic potential for PVR.

### 3.7 Immune infiltration analyses

**FIGURE 6:**
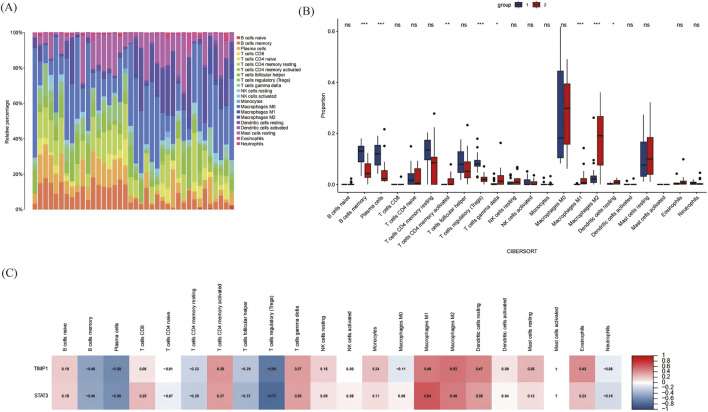
Immune infiltration analyses. **(A)** The histograms of 21 immune cell proportions in PVR samples and normal samples. **(B)** The box plot of differences in immune infiltration in the two groups. *p < 0.05, **p < 0.01, ***p < 0.001. Blue represents the normal group, and red represents the PVR group. **(C)** The correlation between ferroptosis-related key DEGs (FRKGs) expression and different immune cells; the numbers in the cell represent the correlation coefficient. PVR, Proliferative vitreoretinopathy.

We also performed a CIBERSORT immune cell infiltration analysis. The histogram (Figure 6A) shows the proportion of 21 infiltrating immune cells in each sample. There were some differences in immune infiltration between the PVR group and the normal specimen group. Specifically, PVR samples had lower proportions of memory B cells, proportions of plasma cells, and proportions of T regulatory cells; and higher proportions of activated memory CD4^+^ T cells, and proportions of M1-type and M2-type macrophages, compared with normal samples (Figure 6B). In the relationship between the expression of key genes and immune cell infiltration, the expression of TIMP1 and STAT3 was significantly negatively correlated with the proportion of memory B cells, plasma cells, and T regulatory cells (Figure 6C).

### 3.8 Experimental validation

**FIGURE 7:**
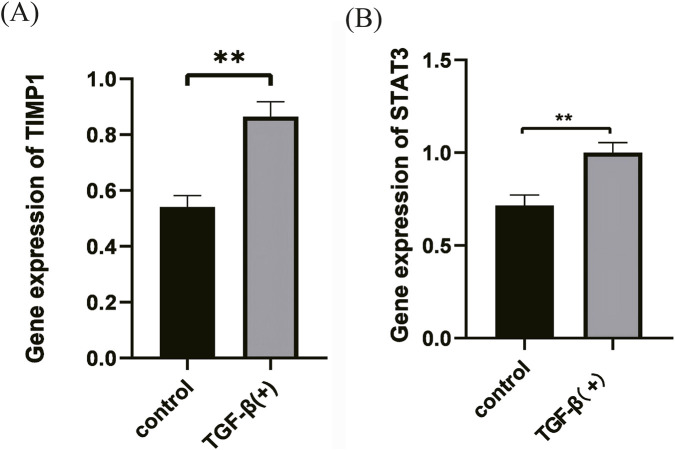
External validation of ferroptosis-related key DEGs (FRKGs). **(A,B)** The mRNA levels of TIMP1 and STAT3 adjusted by GAPDH were evaluated in cell samples by qRT-PCR. Control: normal control group; TGF-(β): the EMT model group, **p < 0.01 vs. TGF-(β).

Key genes were verified using qRT-PCR analysis. The results showed that the expression of both TIMP1 and STAT3 was significantly upregulated and statistically significant (p < 0.05) in the EMT model group compared to the normal group, which was consistent with the results of bioinformatics analysis (Figure 7A,B). Western blot analysis showed significantly increased GPX4 protein expression in retinal tissues from the rabbit PVR model compared with controls (P < 0.001) (Supplementary Figure S1).

## 4 Discussion

Proliferative vitreoretinopathy (PVR) is a vision-threatening retinal disorder characterized by excessive proliferation, migration, and phenotypic transformation of retinal pigment epithelium (RPE) cells following retinal injury (Idrees et al., 2019; Della Volpe Waizel et al., 2024). Ferroptosis, as a novel mode of programmed cell death, is closely associated with the pathophysiological state of various ocular diseases, such as corneal alkali burns, diabetic retinopathy, glaucoma, and age-related macular degeneration (Wang et al., 2022; Guo et al., 2022; Peng et al., 2020). In addition, targeting ferroptosis is a promising therapeutic approach for ocular disease (Wang et al., 2022; Zhang et al., 2022). Although epithelial–mesenchymal transition (EMT), oxidative stress, and inflammation are well-recognized contributors to PVR pathogenesis, the potential involvement of ferroptosis-related pathways remains incompletely understood.

In the present study, we performed a comprehensive transcriptomic analysis combined with machine learning approaches to explore whether ferroptosis-associated gene expression patterns are altered in PVR. Rather than establishing a direct causal role for ferroptosis, our findings suggest that ferroptosis-related regulatory pathways may be engaged during PVR progression and could interact with known pathological processes such as extracellular matrix remodeling and immune microenvironment alterations.

We identified 56 ferroptosis-related DEGs between PVR and normal samples, which were mainly involved in wound healing and tissue remodeling processes during biological processes by GO enrichment analysis. Studies have shown that PVR is a complex pathological process involving an abnormal repair response after retinal injury (Idrees et al., 2019). The formation of PVR is associated with a variety of cell types in the eye, including retinal pigment epithelium (RPE) cells, neuroglia, fibroblasts, fibroblast-like cells, and macrophages. These cells play an important role in the pathogenesis of PVR, especially during retinal injury and repair. And wound healing and tissue remodeling are key aspects in the development of PVR (Feist et al., 2014). During normal wound healing, tissues go through three stages: inflammation, proliferation and remodeling (Talbott et al., 2022). However, in PVR, this process becomes excessive and dysregulated. It has been found that ferroptosis plays a role in the wound healing process, that ferroptosis is involved in the diabetic wound healing process, and that inhibition of ferroptosis ameliorates the inflammatory infiltrate of diabetic wounds and accelerates wound healing through activation of the PI3K/AKT pathway (Li et al., 2021). Therefore, ferroptosis may be involved in the development of PVR by affecting the wound healing process. KEGG pathway enrichment analysis showed that differentially expressed FRDGs were mainly enriched in the ferroptosis pathway, atherosclerosis pathway, and the HIF-1 signaling pathway. The HIF-1 signaling pathway is a key regulator of the cellular response to the hypoxic environment, and consists of two subunits, HIF-1α and HIF-1β (Mialet-Perez and Belaidi, 2024). Under normoxia conditions, HIF-1α is hydroxylated and rapidly degraded, whereas under hypoxic conditions, HIF-1α becomes stable and is able to activate a variety of genes associated with adaptation to hypoxia, such as the VEGF gene, which promotes angiogenesis, and the EPO gene, which regulates erythropoiesis This property of the HIF-1 signaling pathway has led to an important role in a variety of hypoxia-associated disorders, including tumor cardiovascular diseases and some ocular diseases (Guo et al., 2023; Xu et al., 2023). Although direct evidence linking HIF-1 signaling to PVR remains limited, hypoxia-responsive pathways have been implicated in retinal diseases characterized by cellular proliferation and extracellular matrix remodeling. Therefore, activation of HIF-1–related signaling may represent an adaptive or secondary response during PVR progression rather than a primary driver of disease onset.

In this study, TIMP1 and STAT3 were identified as ferroptosis-associated candidate genes through integrative bioinformatics and machine learning analyses. Importantly, both genes are known to participate in multiple biological processes beyond ferroptosis, including extracellular matrix remodeling, inflammation, cell proliferation, and EMT. Therefore, they should not be regarded as ferroptosis-specific markers. TIMP1 is a well-characterized inhibitor of matrix metalloproteinases and plays a crucial role in extracellular matrix turnover and tissue remodeling (Cabral-Pacheco et al., 2020; Tian et al., 2022). Dysregulated extracellular matrix accumulation is a hallmark of PVR, and increased TIMP1 expression may contribute to fibrotic membrane formation by modulating matrix degradation and cellular migration. In addition, TIMP1 has been reported to exert cytokine-like functions and participate in inflammatory regulation, which may further influence PVR progression (Schoeps et al., 2023; Symeonidis et al., 2012). STAT3 is a multifunctional transcription factor involved in cell survival, proliferation, inflammation, and EMT (Sadrkhanloo et al., 2022). Activation of STAT3 signaling has been implicated in EMT-related phenotypic changes of RPE cells, a central event in PVR pathogenesis. Given its broad regulatory roles, increased STAT3 expression in PVR may reflect enhanced cellular activation and inflammatory signaling rather than a direct indication of ferroptotic cell death. Taken together, our results suggest that TIMP1 and STAT3 may represent convergent nodes linking ferroptosis-related regulatory responses with fibrosis- and inflammation-associated pathways in PVR, rather than exclusive mediators of ferroptosis. Future studies need to delve into the specific mechanisms of TIMP1 and STAT3 in ferroptosis in PVR, including how they interact with other molecules and their specific regulatory pathways in lesion development. In addition, investigating their potential as potential therapeutic targets and how to inhibit the development of PVR by interfering with these genes is also an important direction for future research.

ROC curve analysis demonstrated that TIMP1 and STAT3 showed strong discriminative ability between PVR and control samples within the discovery dataset. However, given the limited sample size and the absence of an independent validation cohort, these results should be interpreted with caution. Therefore, TIMP1 and STAT3 should be regarded as preliminary candidate biomarkers rather than definitive diagnostic markers for PVR. Future studies involving larger cohorts and independent validation datasets will be necessary to evaluate their diagnostic robustness and clinical applicability.

The inflammatory response plays a key role in the development of PVR. When detachment or other damage occurs in the retina, inflammatory cells such as macrophages and lymphocytes are attracted to the damaged area. These inflammatory cells release cytokines and growth factors, such as tumor necrosis factor alpha (TNF-α) and platelet-derived growth factor (PDGF), which promote the activation, proliferation, and migration of retinal pigment epithelium (RPE) cells. RPE cells are normally quiescent, but in PVR, cytokine stimulation resulting from inflammatory responses causes RPE cells to undergo epithelial-mesenchymal transformation (EMT) into fibroblast-like cells with the ability to migrate and proliferate. These cells are not only involved in the formation of proliferative membranes, but also enhance the stability and contractility of proliferative membranes by releasing matrix and collagen, which ultimately leads to retinal pulling and detachment. In addition, the inflammatory response may further contribute to the development of PVR by activating other signaling pathways such as NF-κB, MAPK, JAK/STAT, and PI3K/Akt. The activation of these signaling pathways increased the proliferation, migration, and differentiation of RPE cells, which exacerbated the pathological process of PVR (Newman et al., 2015; Duan et al., 2023). Therefore, we analyzed the immune microenvironment by using CIBERSORT to investigate the molecular immune mechanisms associated with ferroptosis in PVR. The results showed that the proportion of memory B cells, the proportion of plasma cells, and the proportion of T regulatory cells were significantly decreased, and the proportion of memory CD4^+^ T cells, and the proportion of M1-type and M2-type macrophages were significantly increased in PVR samples. This is consistent with previous findings (Charteris et al., 1993). Unsurprisingly, the high expression of TIMP1 and STAT3 was associated with a lower proportion of memory B cells and a lower proportion of plasma cells in the PVR. We hypothesize that these two FRKGs are involved in the inflammatory response and immune processes of PVR onset and progression by influencing the immune microenvironment. It should be noted that immune infiltration was inferred from bulk transcriptomic data using computational deconvolution, which has inherent limitations. Therefore, the observed immune alterations require further validation using experimental and single-cell approaches.

Our qRT-PCR results showed an increase in the expression of TIMP1 and STAT3 with statistical significance (P < 0.05). In the present study, Western blot analysis demonstrated significantly increased GPX4 protein expression in retinal tissues from the rabbit PVR model compared with controls (P < 0.001). Notably, increased GPX4 expression does not necessarily indicate the decrease of ferroptotic cell death. Instead, it may represent a compensatory or protective response to elevated oxidative stress and lipid peroxidation during PVR progression. This finding supports the notion that ferroptosis-related regulatory mechanisms are altered in PVR, although further functional studies are required to determine whether ferroptosis directly contributes to cellular injury in this context.

In summary, this study provides a transcriptome-based exploration of ferroptosis-associated gene expression patterns in PVR. Our findings suggest that ferroptosis-related regulatory pathways may interact with extracellular matrix remodeling, EMT, and immune responses during PVR progression. However, this study is limited by the use of a single public dataset, a relatively small sample size, and the lack of direct functional manipulation of ferroptosis. Future *in vivo* and *in vitro* studies will be required to clarify the mechanistic role of ferroptosis and to validate the clinical relevance of TIMP1 and STAT3 in PVR.

## 5 Conclusion

In conclusion, this study provides a transcriptome-based exploration of ferroptosis-associated gene expression patterns in PVR. TIMP1 and STAT3 were identified as candidate genes associated with ferroptosis-related regulatory pathways and may reflect pathological processes involving extracellular matrix remodeling and immune responses in PVR. However, the mechanistic role of ferroptosis in PVR remains to be clarified, and further experimental and clinical validation is required.

## Data Availability

The transcriptomic data analyzed in this study are publicly available in the NCBI Gene Expression Omnibus (GEO) repository under accession numbers GSE28133 and GSE285957. All scripts used for data preprocessing and analysis are available at https://github.com/liujiangying68/PVR-ferroptosis-transcriptomics.
