# Supplementary material for: Identification of potential ferroptosis-related biomarkers in proliferative vitreoretinopathy based on machine learning
Source: Front Mol Biosci. 2026 Feb 10;13:1725407. doi: 10.3389/fmolb.2026.1725407 (PMC12930359; doi:10.3389/fmolb.2026.1725407)
Supplement: Supplementary file 1 [file Image1.pdf]

Supplementary

Figure 1S Relative GPX4 protein expression (normalized to GAPDH).

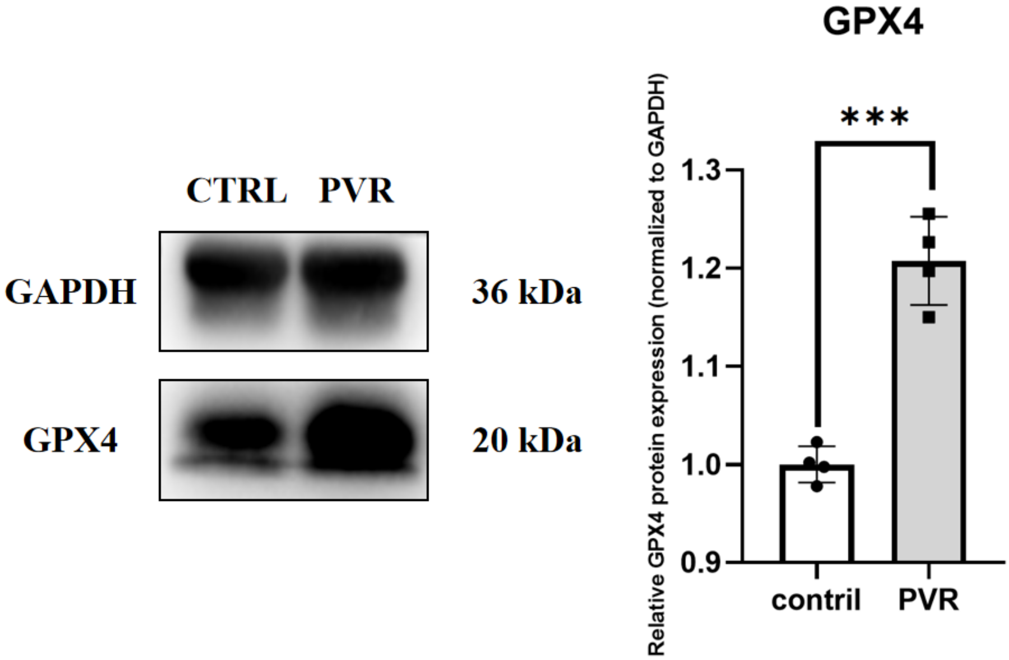

The protein levels of GPX4 adjusted by GAPDH were evaluated in rabbits samples by western blotting. *n*=4, ***p<0.001.
